# An assessment of skill erosion on high‐dose rate brachytherapy treatment planning

**DOI:** 10.1002/acm2.70300

**Published:** 2025-10-14

**Authors:** Dominic J. DiCostanzo, Theodore T. Allen, Ahmet S. Ayan, Allison Quick, Kevin D. Evans, Emily S. Patterson

**Affiliations:** ^1^ School of Health and Rehabilitation Sciences The Ohio State University Columbus Ohio USA; ^2^ Department of Radiation Oncology Wexner Medical Center The Ohio State University Columbus Ohio USA; ^3^ Department of Integrated Systems Engineering The Ohio State University Columbus Ohio USA

**Keywords:** Gauge R&R analysis, high‐dose rate brachytherapy, skill erosion, treatment planning variability, quality improvement

## Abstract

**Purpose:**

To assess skill erosion for physicists after leaving brachytherapy service and contributors to variability during brachytherapy treatment planning.

**Methods:**

Medical physicists simulated planning for nine patients by creating treatment plans twice, 2 months apart. The physicists were stratified as “On‐Service” if they were assigned to and actively participating in the brachytherapy service (including treatment planning) during the study or “Off‐Service” if they were not assigned, were not actively engaged in the service, and had not performed treatment planning the 6 months prior to the study commencement or during the study. A mixed effects model with Bonferroni correction was used to test for statistical significance between the stratified groups and Cohen's D was used to compare the effect of skill erosion. ANOVA analysis of a crossed Gauge Repeatability and Reproducibility (Gauge R&R) study quantified three contributors to variability for eight segments of the planning process: [1] repeatability (intra‐observer), [2] reproducibility (inter‐observer), and [3] patient‐to‐patient variation. Process capability was deemed acceptable when the Total Gauge R&R was less than 10%, marginally acceptable between 10% and 30%, and unacceptable when greater than 30%.

**Results:**

The group of On‐Service study participants had lower variability on 10 of 19 measures in the brachytherapy planning process than Off‐Service study participants with the effect size being small, Cohen's D between 0.15 and 0.30 for significant measures. This indicates skill erosion does affect physicists after leaving the brachytherapy service for a period of 6 months or more. For both groups, only the bladder D2cc metric had a controlled amount of variability other than applicator reconstruction.

**Conclusions:**

Brachytherapy treatment planning suffers from skill erosion. Physicists should seek to mitigate its effects and evaluate competency on an ongoing basis.

## 1 INTRODUCTION

Specialized skills require regular use to maintain proficiency. According to the American Association of Physicists in Medicine (AAPM), the frequency at which medical physicists perform routine duties can directly impact their competency.^1^ Evidence elucidates that cognitive skills decay faster than physical skills.^2^

The majority of tasks that qualified medical physicists (QMPs) perform require cognitive skills.^3^ In smaller practices, the exposure to certain tasks may be limited due to the patient population treated. There may also be a reduction in exposure at larger practices due to operational requirements such as limited rotations of staff or the necessary specialization of team members. Regardless of practice environment, unless there is concerted effort to interrupt skill decay, there is a strong probability that QMPs will suffer from its effects. The probability of skill loss increases for procedures that are less common. One such area that physicists may participate less frequently in is brachytherapy. The number of brachytherapy cases is decreasing.^4^ As cases decline, physicians and physicists will face increased skill erosion.

Treatment planning is a cognitive skill and one of the key activities within brachytherapy services that QMPs may realize skill decay. Regardless of the contributing factors, the impact of diminished skills on patients is direct. To combat this, the AAPM has generated a framework for a competency and credentialing program including a recommendation of observational competency assessment with feedback at least four times per year for brachytherapy procedures.^1^

To assess the impacts of skill erosion on brachytherapy treatment planning, we performed a Gauge Repeatability and Reproducibility (Gauge R&R) study with simulated brachytherapy treatment planning tasks. Repeatability refers to intra‐observer variability or the variation of the same individual performing the same task multiple times. Whereas reproducibility refers to inter‐observer variability or the variation between individuals performing the same task. Gauge R&R also quantifies the patient‐to‐patient variability to assess its impact on the variability of the process.

## 2 METHODS AND MATERIALS

### 2.1 Patient and participant identification

This study was approved by our Institutional Review Board. Nine patients previously treated with the ring and tandem (R&T) applicator were randomly selected; none of which were treated with a hybrid applicator (i.e., needles in addition to the rigid applicator) [Table 1]. The data for each patient was anonymized, the order randomized, and the patients were anonymized for a second time creating two series of nine patients for each participant. Twelve medical physicists (QMPs and medical physics residents) participated in the study [Table 2]. All physicists were deemed competent in brachytherapy treatment planning during their tenure at our institution. Each physicist had 18 datasets to generate treatment plans on and each dataset included CT datasets for treatment planning and applicator reconstruction, MRI datasets with clinical image registration, and clinical segmentations of the high‐risk CTV (HR‐CTV)^5^ and organs‐at‐risk (OARs).

**TABLE 1:** Patient characteristics.

Age at Treatment		EBRT + Brachytherapy	
Median [Range] (y)	49 [29–87]	Yes	9
		No	0
**ICD‐O Site Code**			
C53.1 ‐ Exocervix	3	**Brachytherapy Applicator**	
C53.9 ‐ Cervix uteri	6	Ring + 6 cm Tandem	9
**FIGO Stage**		**Prescribed Fractional Dose**	
IB	3	550cGy	8
IIB	3	600cGy	1
IIIB	2		
IIIC	1		

**TABLE 2:** Participant characteristics. All physicists in the study have participated in the brachytherapy service during their tenure at our institution.

**Age**		**Highest Degree**	
Median [Range] (y)	34 [26–57]	MS	8
		DMP	2
**Sex**		PhD	2
M	8		
F	4	**Years of Experience**	
		Median [Range]	7.3 [1.5–19]
**Brachytherapy Service Participation**			
On‐Service	5	**Board Certification**	
Off‐Service	7	Yes	10
		No	2
**Participated in Brachytherapy Service at OSU**			
Yes	12	**Length of Board Certification**	
No	0	Median [Range] (y)	6 (0–15)
**Completed Residency at OSU**		**Physicist Status**	
Yes	6	QMP	10
No	6	Resident	2

### 2.2 Generation of treatment plans

As a simulated planning study, the participants were asked to follow our institutional guidelines for manual reconstruction of the applicator and were encouraged to review the guidelines and procedures if they desired. Participants were then asked to generate treatment plans for a single fraction with a 550cGy prescription dose irrespective of the treated clinical prescription.^6^ The plans created were to be of sufficient quality that the participants would ask for physician review and approval in a clinical setting. The plans were generated in Eclipse treatment planning system (Brachytherapy Treatment Planning v16.1) using the GammaMedplus afterloader, a step size of 5 mm, and nominal 10 Ci source strength.

Our institution uses volumetric‐based treatment planning following consensus recommendations.^5^, ^6^, ^7^, ^8^, ^9^ The dose metrics used for plan evaluation included the dose covering at least 90% of the HR‐CTV (D90%) and the minimum dose to the hottest 2cc for each OAR (D2cc). Each participant was given the first nine datasets in September 2022 and asked to generate the treatment plans. The second nine datasets were provided in November 2022.

### 2.3 Export of quantitative data

Dosimetric metrics were extracted via automated methods and stored as comma‐separated value (CSV) files for analysis. The DICOM Radiotherapy Plan (RTPlan) and Radiotherapy Structure Set (RTStruct) files were processed using Python. From the RTPlan file, the control points were extracted for each channel of the R&T applicator that defines the (X, Y, Z) coordinates of the dwell position and cumulative dwell time. In addition, the source dwell times were calculated from the RTPlan file. The total dwell time and the number of control points each participant used were also obtained. From the RTStruct file, the number of contour points used to reconstruct each channel of the R&T and the (X, Y, Z) coordinates of each point used in the reconstruction.

### 2.4 Polynomial fits

Unlike anatomical segmentations the reconstructed applicator cannot have metrics like the Dice coefficient or intersection over union calculated for comparison as they cannot be converted to segmentation masks. In addition, the number of points used by the participants varied for both the tandem and ring. As a result, the contours for the applicator were fit with a polynomial until the point after the final dwell position. (Details available in the supplemental information.) The fit coefficients were then evaluated using Gauge R&R to determine the variability in applicator reconstruction.

### 2.5 Data analysis

We used a crossed Gauge R&R design with analysis of variance (ANOVA). The planners were stratified into two groups, “On‐Service” if they were assigned to and actively participating in the brachytherapy service (including treatment planning) during the study or “Off‐Service” if they were not assigned, were not actively engaged in the service, and had not performed treatment planning the 6 months prior to the study commencement or during the study. The standard criterion for acceptability determination is the percentage of all variation resulting from total R&R (%GRR). The system is considered acceptable if the %GRR is less than 10%. If the %GRR is between 10% and 30%, it is considered marginally acceptable; if the %GRR exceeds 30%, it is considered unacceptable.^10^ We assessed the variation in planned dose to three OARs and HR‐CTV, dose to Manchester System reference points (A left, A right), Internation Commission on Radiation Units and Measurement Report 38 points (rectum, and bladder), dwell time of the applicator components (ring and tandem, respectively), and the reconstruction using the fit coefficients. Minitab statistical analysis software (Minitab, LLC, Coventry, UK, https://www.minitab.com/) was used to calculate the R&R for each data type using ANOVA.

The sum of squared differences for each plan, patient, planner, and repetition were computed directly from the data provided. The sum of squared differences for the patient by planner interactions was computed as:

(1) $$SS_{pat\cdot planner}=SS_{tot}-SS_{pat}-SS_{planner}-SS_{rep}$$

where $SS_{tot}$ is the sum of squared differences for each plan. The Gauge R&R value is computed using the calculated variance for repetitions and planners:

(2) $$\%GRR=\frac{{\sigma^{2}}_{rep}+{\sigma^{2}}_{planner}}{{\sigma^{2}}_{tot}}\times 100$$

The controllable variation of the system is given by the repeatability $\left({\sigma^{2}}_{rep}\right)$ and reproducibility $\left({\sigma^{2}}_{planner}\right)$ components of %GRR. The uncontrollable variation is given by the patient‐to‐patient variation $\left({\sigma^{2}}_{pat}\right)$ calculated by:

(3) $${\sigma^{2}}_{pat}=\frac{MS_{pat}-MS_{pat\cdot planner}}{r\cdot p}$$

where $MS_{pat}$ is the mean square of the patient, $MS_{pat\cdot planner}$ is the mean square of the patient‐planner interaction, r is the number of repetitions, and p is the number of patients.

To evaluate statistical significance between the stratified groups, a mixed effects model (MEM) was created. As the plans were generated two times, the original plan number (i.e., 1–9) was assigned to the random factors, with trial (1 or 2) and planner service status (0 for Off‐Service and 1 for On‐Service) assigned to the fixed factors. The trial number was also nested within the original plan number to include repeatability in the evaluation of statistical significance.

### 2.6 Physician review

One (1) board‐certified physician with expertise in gynecological brachytherapy rated the plans using a 3‐point scale commonly employed in other studies: (1) clinically acceptable, would approve for treatment, (2) minor modifications necessary, would request minor adjustments before approving for treatment, (3) clinically unacceptable, state of the current plan is unsuitable for delivery and would require multiple changes. Due to the number of plans created by the planners and the time required for review, 4 plans were randomly selected and presented from each of the 12 planners (48 of 216 plans). The physician was blinded to the identity of the treatment planners and provided only the patient identification information to facilitate access to the plans in the TPS.

## 3 RESULTS

**FIGURE 1:**
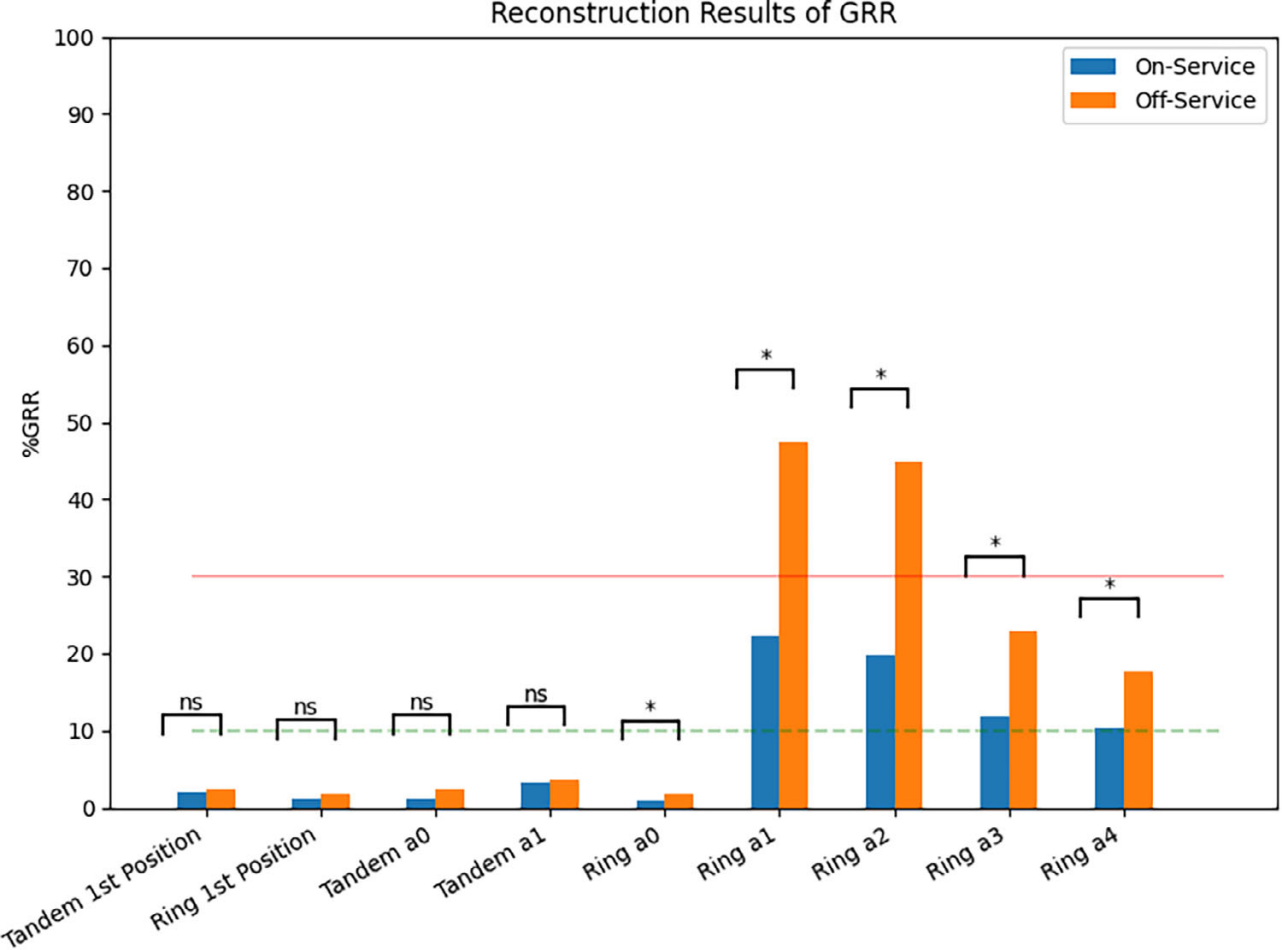
Clustered column of the metrics used to evaluate reconstruction of the tandem and ring by group. Brackets above each pair display the statistical significance of the difference between the groups for the given metric. Significance set to *p*‐value < 0.00263 (Bonferroni correction). The dashed line at 10% GRR is the value below which a metric is deemed to be acceptable by Gauge R&R. The 30% GRR line shows the line of acceptability. Tandem 1^st^ Position and Ring 1^st^ Position represent the variability in the first selected point of the applicator. The “a” values represent the variability of the fit coefficients for the polynomial fit of the applicator, which acts a surrogate for variability in the location of the applicator.

**FIGURE 2:**
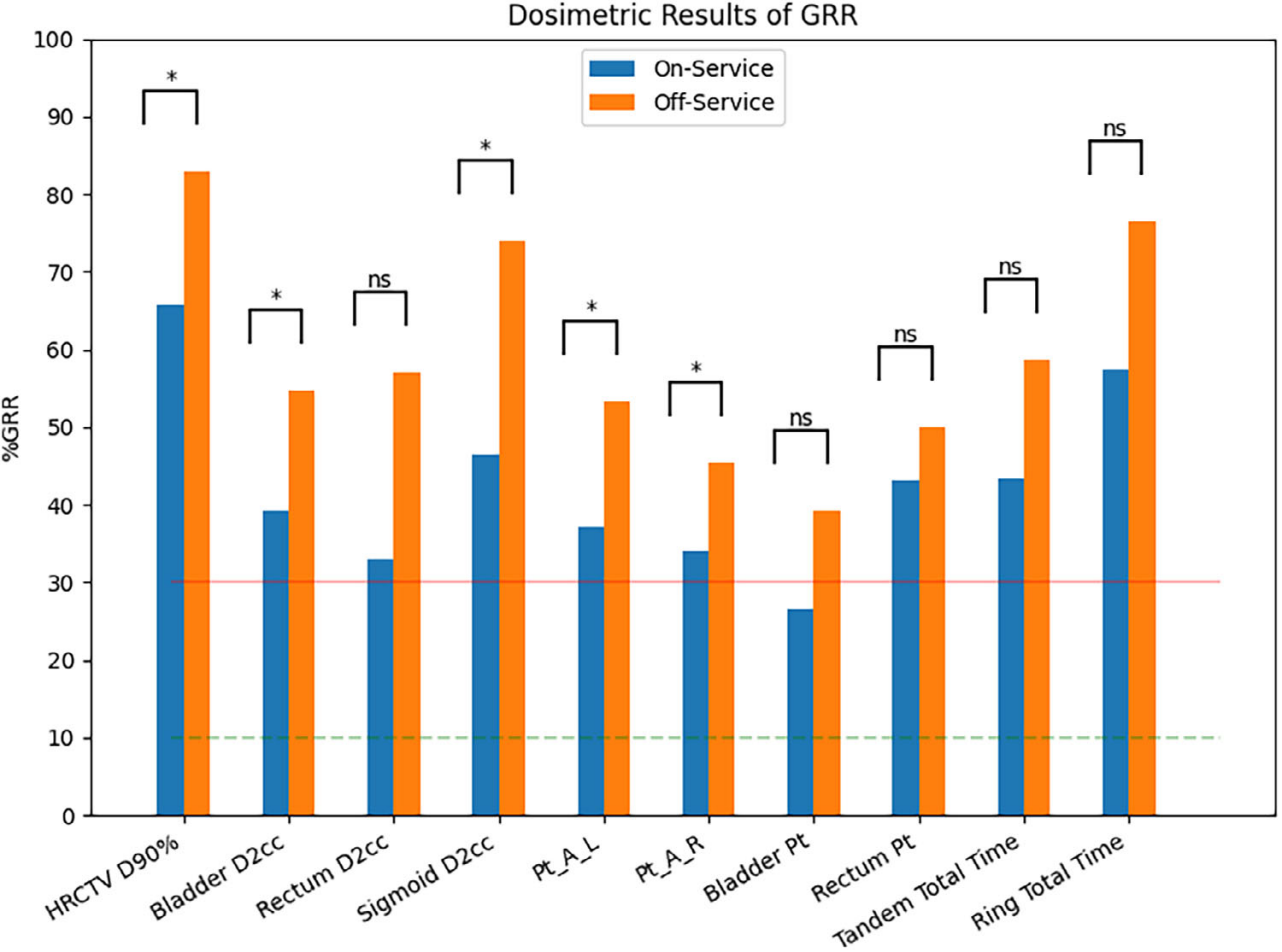
A clustered column of the dosimetry related attributes evaluated. Pt_A, Bladder Pt, and Rectum Pt represent the Manchester System points as inserted in the clinical treatment plan. The locations of the points from the treated plan were extracted and applied to the plans generated during the study. The dashed line at 10 %GRR is the value below which a metric is deemed to be acceptable by Gauge R&R. The 30 %GRR line shows the line of acceptability. Brackets above each pair display the statistical significance of the difference between the groups for the given metric. Significance set to *p*‐value < 0.00263 (Bonferroni correction).

Across all evaluated metrics, the group of On‐Service study participants displayed less variation than the group of Off‐Service study participants [Figures 1 and 2]. Of the 19 evaluated metrics, 10 were statistically significant, indicating that those who were On‐Service performed planning with less variability than those who were Off‐Service. Cohen's D showed that each of the metrics with statistically significant variability had a small effect with values between 0.15 and 0.30. For the On‐Service group, the only metric that showed a controlled amount of variation was the bladder reference point (26.5 %GRR) other than the reconstruction of the applicator.

### 3.1 Applicator reconstruction

The brachytherapy treatment planning system uses the first contour point as the applicator's physical tip. Any errors in identifying the tip will directly result in shifts to all planned dwell positions and can affect the planned dose distribution in all three dimensions (cranial‐caudal, left‐right, and anterior‐posterior). In Table 3, the results indicate that the first contour point for the tandem and ring are well controlled, and the MEM indicated that service status had no effect on its selection [Figure 1].

**TABLE 3:** The Gauge R&R results analyzed using ANOVA applied to metrics related to applicator reconstruction by group. The Total Gauge R&R represents the amount of variability in the study that is attributable to repeatability and reproducibility. Patient‐To‐Patient variability represents the variability in the study that is attributable to the differences between patients on which the planning was simulated. Tandem First Point and Ring First Point refer to the first point of the reconstruction. The “a” values represent the coefficients of the polynomial fit which represents the physical location of the reconstructed applicator.

On‐Service	Percent Study Variance (%) (95% CI)				
Variance Components	Tandem First Point	Ring First Point	Tandem a0	Tandem a1	
**Total Gauge R&R**	2.0 (1.0–3.6)	1.1 (0.6–1.7)	1.2 (0.6–2.8)	3.3 (1.7–5.1)	
** Repeatability**	1.8 (0.9–2.7)	1.0 (0.5–1.6)	0.9 (0.5–1.3)	2.6 (1.4–4.2)	
** Reproducibility**	0.9 (0.4–2.9)	0.3 (0.0–1.0)	0.8 (0.4–2.6)	1.9 (0.6–3.6)	
**Patient‐To‐Patient**	100.0 (99.9–100.0)	100.0 (100.0–100.0)	100.0 (100.0–100.0)	100.0 (99.9–100.0)	

Off‐Service	Percent Study Variance (%) (95% CI)				
Variance Components	Tandem First Point	Ring First Point	Tandem a0	Tandem a1	
**Total Gauge R&R**	2.5 (1.2–5.6)	1.9 (1.0–3.6)	2.5 (1.2–5.6)	3.6 (1.9–5.5)	
** Repeatability**	0.9 (0.5–1.4)	1.3 (0.7–2.0)	0.9 (0.5–1.4)	3.6 (1.9–5.5)	
** Reproducibility**	2.3 (1.1–5.5)	1.3 (0.6–3.2)	2.3 (1.1–5.5)	0.0 (0.0–1.7)	
**Patient‐To‐Patient**	100.0 (98.9–100.0)	100.0 (99.9–100.0)	100.0 (99.8–100.0)	99.9 (99.9–100.0)	

On‐Service	Percent Study Variance (%) (95% CI)				
Variance Components	Ring a0	Ring a1	Ring a2	Ring a3	Ring a4
**Total Gauge R&R**	1.0 (0.5–1.9)	22.3 (11.7–33.8)	19.7 (10.3–29.9)	11.8 (6.2–18.3)	10.4 (5.4–16.4)
** Repeatability**	0.8 (0.4–1.3)	17.2 (9.0–26.6)	15.6 (8.1–24.2)	9.5 (4.9–14.8)	7.8 (4.0–12.3)
** Reproducibility**	0.5 (0.2–1.6)	14.1 (5.9–24.7)	12.0 (4.5–21.5)	7.1 (2.5–13.4)	6.8 (2.3–13.1)
**Patient‐To‐Patient**	100.0 (100.0–100.0)	97.5 (94.1–99.3)	98.0 (95.4–99.5)	99.3 (98.3–99.8)	99.5 (98.6–99.9)

Off‐Service	Percent Study Variance (%) (95% CI)				
Variance Components	Ring a0	Ring a1	Ring a2	Ring a3	Ring a4
**Total Gauge R&R**	1.7 (0.9–3.2)	47.4 (26.1–73.5)	44.9 (24.5–70.8)	23.0 (12.1–39.4)	17.7 (9.3–28.1)
** Repeatability**	1.1 (0.6–1.7)	30.2 (16.9–42.2)	29.1 (16.1–40.9)	17.9 (9.4–26.5)	15.7 (8.2–23.5)
** Reproducibility**	1.3 (0.6–2.9)	36.6 (18.2–67.9)	34.2 (16.9–65.1)	14.5 (6.6–33.4)	8.0 (3.4–20.0)
**Patient‐To‐Patient**	100.0 (100.0–100.0)	88.0 (67.8–96.5)	89.4 (70.6–97.0)	97.3 (91.9–99.3)	98.4 (96.0–99.6)

For the QMPs who are On‐Service, the digitization of the applicator parts is well‐controlled for the tandem (range of 1.2%–3.3% GRR) and marginally controlled for the ring (range of 1.0%–22.3% GRR). Those who were Off‐Service also reconstructed the tandem in a well‐controlled manner (range of 2.5%–3.6% GRR) [Table 3]. However, the ring reconstruction was not controlled (range of 1.7%–47.4% GRR). The MEM indicates that service status has no effect on tandem reconstruction but does impact ring reconstruction [Figure 1].

### 3.2 Dosimetry metrics

**FIGURE 3:**
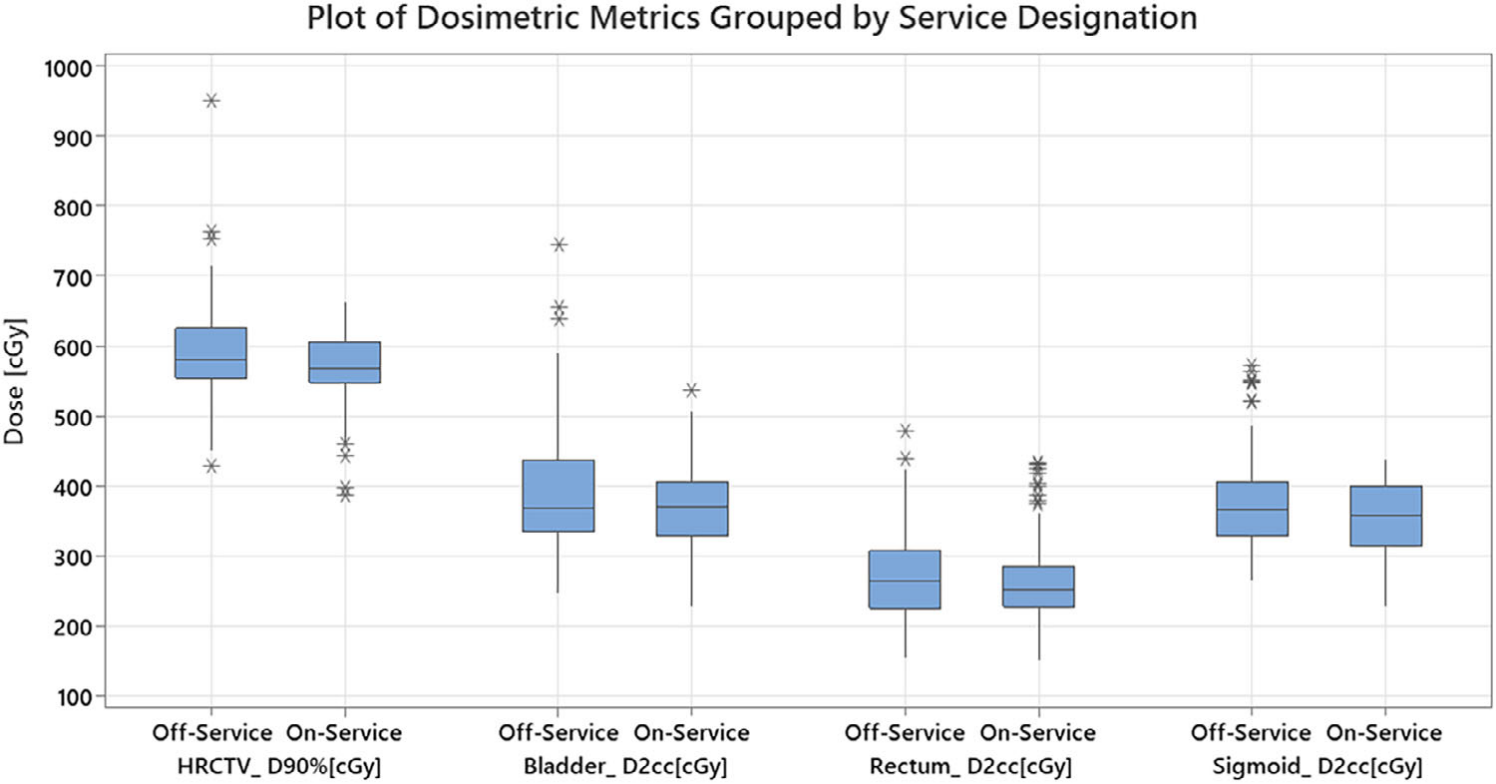
A boxplot of the dosimetric metrics evaluated with the differences between the Off‐Service and On‐Service groups present. The shaded box indicates the first through third quartiles with the line indicating the median. The individual marks above and below indicate outliers.

**FIGURE 4:**
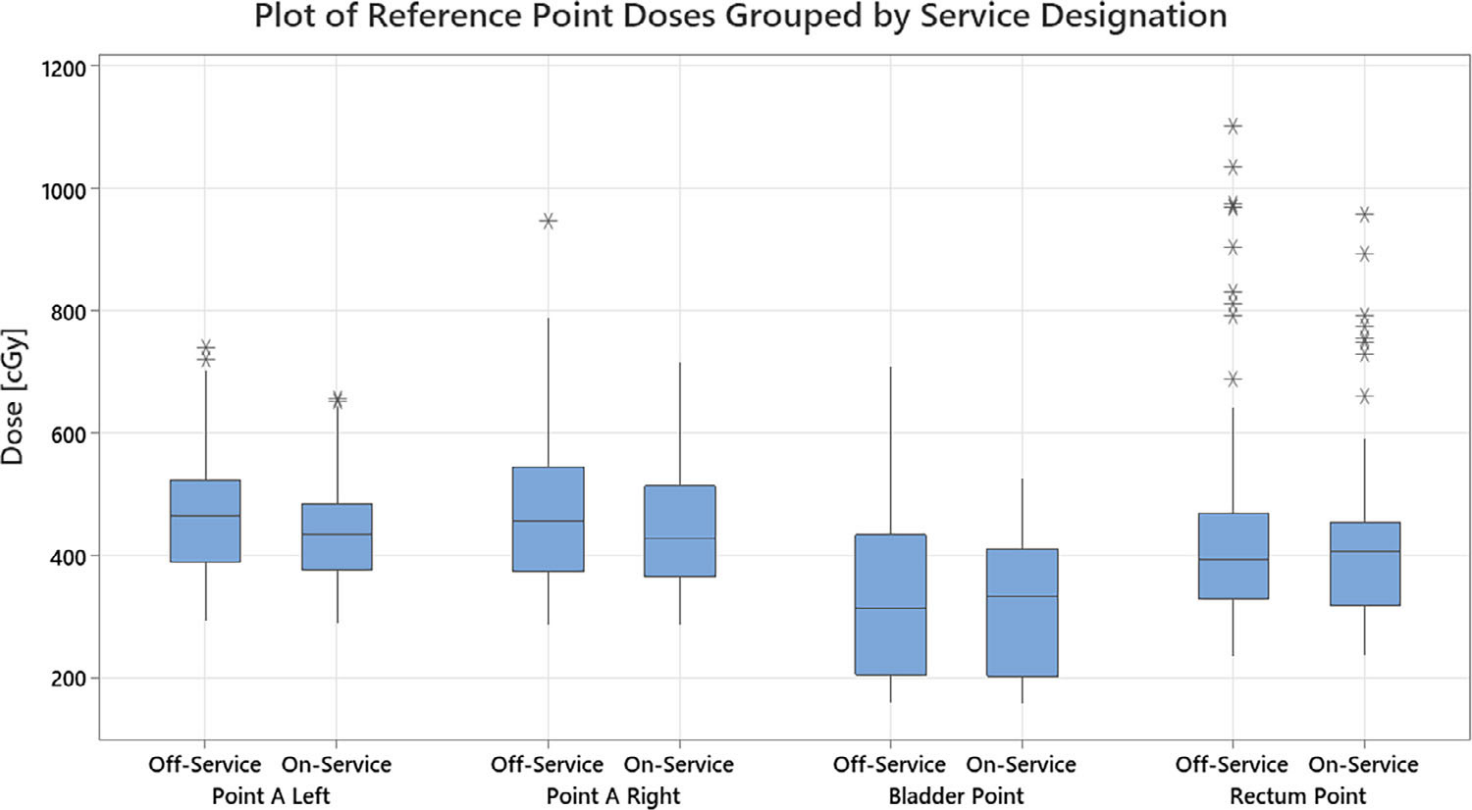
A boxplot of the reference point doses evaluated with the differences between the Off‐Service and On‐Service groups present. The shaded box indicates the first through third quartiles with the line indicating the median. The individual marks above and below indicate outliers.

The dosimetric metrics and reference points [Figures 2, 3, and 4] show that plan creation is highly variable regardless of service status [Table 4]. Only the bladder reference point was marginally controlled for the On‐Service group; no metrics were controlled for Off‐Service. The MEM suggests Off‐Service physicists show significantly greater variability than those On‐Service, except for the bladder and rectum reference points and the rectum D2cc metric [Figure 2].

**TABLE 4:** The Gauge R&R results analyzed using ANOVA applied to treatment plan and dosimetry metrics by group. The Total Gauge R&R represents the amount of variability in the study that is attributable to repeatability and reproducibility. Patient‐To‐Patient variability represents the variability in the study that is attributable to the differences between patients on which the planning was simulated. Point A Left/Right, Bladder, and Rectum points represent the Manchester System and ICRU dosimetric points as used in the treated clinical plan.

On‐Service	Percent Study Variance (%) (95% CI)				
Variance Components	HR‐CTV D90%	Point A Left	Point A Right	Tandem Total Time	Ring Total Time
**Total Gauge R&R**	65.8 (40.0–87.2)	37.2 (20.1–58.7)	33.9 (18.1–56.2)	43.3 (23.9–67.9)	57.5 (33.7–77.3)
** Repeatability**	39.0 (22.5–53.5)	21.3 (11.4–31.8)	16.8 (8.9–53.1)	37.4 (20.6–53.7)	53.6 (31.1–70.6)
** Reproducibility**	53.0 (30.2–81.5)	30.5 (15.8–54.0)	29.4 (15.3–53.1)	21.8 (9.2–57.5)	20.9 (6.6–58.0)
**Patient‐To‐Patient**	75.3 (49.0–91.7)	92.8 (81.0–98.0)	94.1 (82.8–98.3)	90.2 (73.4–97.1)	81.8 (63.5–94.2)

Off‐Service	Percent Study Variance (%) (95% CI)				
Variance Components	HR‐CTV D90%	Point A Left	Point A Right	Tandem Total Time	Ring Total Time
**Total Gauge R&R**	82.3 (59.2–94.4)	53.2 (30.3–77.0)	45.4 (25.1–69.9)	58.7 (34.4–81.6)	76.4 (51.1–91.3)
** Repeatability**	61.2 (38.0–75.1)	37.7 (21.5–51.3)	25.8 (14.2–36.8)	40.6 (23.6–54.3)	57.6 (35.7–71.7)
** Reproducibility**	55.9 (34.1–83.8)	37.5 (19.0–68.9)	37.4 (19.5–65.2)	42.4 (22.1–73.8)	50.2 (28.8–80.0)
**Patient‐To‐Patient**	56.0 (33.0–80.6)	84.7 (63.8–95.3)	89.1 (71.5–96.8)	81.0 (57.8–93.9)	64.5 (40.9–86.0)

On‐Service	Percent Study Variance (%) (95% CI)				
Variance Components	Bladder D2cc	Rectum D2cc	Sigmoid D2cc	Bladder Point	Rectum Point
**Total Gauge R&R**	39.1 (21.3–63.1)	32.9 (17.7–51.8)	46.4 (25.9–68.8)	26.5 (14.0–44.8)	43.1 (23.8–63.9)
** Repeatability**	28.2 (15.2–41.7)	26.1 (13.9–39.2)	35.8 (19.7–51.6)	20.4 (10.7–31.2)	31.9 (17.4–46.6)
** Reproducibility**	27.2 (12.7–56.3)	20.0 (7.6–42.9)	29.4 (12.5–58.8)	16.9 (6.9–38.6)	28.9 (13.5–54.3)
**Patient‐To‐Patient**	92.0 (77.6–97.7)	94.4 (85.5–98.4)	88.6 (72.6–96.6)	96.4 (89.4–99.0)	90.3 (76.9–97.1)

Off‐Service	Percent Study Variance (%) (95% CI)				
Variance Components	Bladder D2cc	Rectum D2cc	Sigmoid D2cc	Bladder Point	Rectum Point
**Total Gauge R&R**	54.7 (31.5–77.6)	65.9 (33.4–77.4)	74.0 (48.1–91.0)	39.2 (21.5–59.2)	50.0 (27.8–66.7)
** Repeatability**	60.7 (17.5–42.5)	46.5 (27.1–61.5)	41.4 (24.7–54.7)	25.9 (14.0–37.5)	60.1 (16.9–42.5)
** Reproducibility**	45.3 (24.8–72.2)	32.8 (16.3–63.6)	61.3 (37.1–85.6)	29.4 (15.1–51.8)	40.0 (21.1–55.5)
**Patient‐To‐Patient**	83.7 (63.1–94.9)	82.3 (63.3–94.3)	67.3 (41.4–87.7)	92.0 (80.6–97.7)	86.6 (74.6–96.1)

Variability in bladder filling impacts the dose delivered to the patient. Compared to Off‐Service physicists, the On‐Service group produced more consistent plans with lower variability, particularly for the bladder D2cc metric. Greater variability can often require more plan modifications or iterations. Since bladder volume can change rapidly, efficient and precise planning is essential to ensure the imaging used for treatment accurately reflects patient anatomy.

### 3.3 Clinical acceptability

A review of the clinical acceptability as rated by the physician indicates that the physicists created treatment plans appropriate for treatment most of the time, 38/48 (79%). Only 13/48 (19%) of the plans generated would require minor modifications; almost exclusively related to changes in HR‐CTV coverage. One plan was deemed clinically unacceptable due to the sigmoid dose and over coverage of the HR‐CTV. The On‐Service group had 17/20 (85%) of the plans generated deemed clinically acceptable as opposed to 21/28 (75%) of the plans by the Off‐Service group.

## 4 DISCUSSION

In this study, we have identified that skill erosion does occur for medical physicists in brachytherapy treatment planning. Using the crossed Gauge R&R method with ANOVA analysis, variability in multiple aspects of the treatment planning process was isolated between physicists who were stratified between On‐Service and Off‐Service groups. The results show that physicists who are On‐Service had less controllable variability than those who were Off‐Service. Even when the controllable variability exceeded the GRR tolerance (30 %GRR) for those On‐Service, the variability encountered was significantly less (10 of 19 metrics) than that of those who were Off‐Service. To our knowledge, this is the first study to investigate skill erosion in radiation therapy treatment planning. At a time when artificial intelligence is gaining popularity across all domains, physicists must be aware of the dangers of skill erosion and learn to mitigate its effects.

Consequentially, it was identified that skill erosion could be a factor in overall plan quality. While almost all dosimetric metrics showed uncontrolled variability between planners, the variability of the On‐Service group was significantly lower than the variability of the Off‐Service group except for the rectum metric and rectum and bladder reference points. In addition, when reviewing the plan scores as performed by the physician, the On‐Service group had 85% (17/20) of their plans scored as clinical compared with 75% (21/28) of the Off‐Service group.

Although the dosimetry related metrics were largely uncontrolled and highly variable, applicator reconstruction showed mixed results. The first point of the applicator reconstruction is well‐controlled for both the ring and tandem. The identification of the first point of the ring and tandem is performed by determining the change in Hounsfield Units between soft tissue and the applicator using the line profile tool within the TPS. The tandem reconstruction was the only well‐controlled process between both groups. Given that the tandem reconstruction is trivial and can be often performed using two points, this is expected. The ring reconstruction was marginally controlled within the On‐Service group, as contrasted with the Off‐Service group where it is uncontrolled. While the reconstruction of the ring is more complicated than the tandem given the physical geometry, the reconstruction process is the same. This indicates that skill erosion could be a contributing factor in the increased variability.

The decreased controllable variability indicates that the brachytherapy treatment planning process can be positively impacted through the introduction of quality improvement measures. While the selection of the first point of the applicator is well controlled for the evaluated applicator, this may not be the case for other applicators with less identifiable features (e.g., needles). Extending this work to other applicators would provide a more comprehensive understanding of the treatment planning process. In addition, while statistically significant, the variability identified in the applicator reconstruction is not likely to be clinically significant. The identified uncertainty is relatively small compared with other uncertainties in brachytherapy treatment planning, such as segmentation or biological processes like internal organ motion. However, this should not preclude a reduction of uncertainty wherever possible.

The variability in the dosimetric metrics could lead to increased iterations during the planning process. If reduced, it could lead to meaningful positive impacts on patients’ clinical care. Specifically, the D2cc metric for the bladder was the only dosimetric metric that was below the acceptability cutoff of 30%. Given the biological process of bladder filling, the faster a plan is generated influences how representative the planning image is of the patient state at treatment. More generally, the physician often caps the number of iterations in brachytherapy as they balance the clinical benefit of the treatment against various risk factors, for example, time under anesthesia. Reducing variability in the initial plan quality presented to the physician for approval could have positive implications for the safety and efficiency of the brachytherapy treatment planning process. It is important to note that the physician would have approved most of the plans and deemed them clinically acceptable. As such, the variability of the plans generated could directly impact the patient via a reduction of dose to OARs in the initial plans presented to the physician. Reducing variability in this regard would require more specific directives and overall guidance provided by the physician than is currently provided at our institution.

The findings of this study emphasize the importance of competency evaluations for QMPs involved in brachytherapy treatment planning. The identified skill erosion can have direct implications for patient care. Patients who experience variations in dose delivery could suffer from suboptimal outcomes. Additionally, we identified that the increased variability associated with Off‐Service group required modifications leading to additional plan iterations, which unnecessarily lengthens the planning process. This not only impacts patient comfort and safety but also strains resources, especially in environments with limited resources.

This study was a simulated treatment planning study, and as such did not represent the clinical pressures often associated with planning. A limitation of this study is that the plans were only repeated twice. However, statistical significance via MEM was achieved suggesting that the findings would not change if we had repeated the plans for a third time. Due to convenience, this study sampled 12 physicists at a single institution, two of which were residents. However, the residents included had completed a brachytherapy rotation and been deemed competent to independently perform brachytherapy treatment planning limiting any potential impact on the results. Further work could look at a broader demographic across multiple institutions. Finally, an attempt to quantify the rate of skill erosion that was uncovered in this study would benefit the field by allowing interventions to be generated at specific times rather than arbitrarily identified points. The current evidence does support the use of competency‐based evaluations in medical physics practice. Practice or simulation prior to clinical work for a skill that has not been dormant could mitigate its effects as suggested in previous literature.^11^

## 5 CONCLUSION

Brachytherapy treatment planning performed by medical physicists suffers from skill erosion. Indicating that the frequency of performing brachytherapy cases significantly affects a medical physicist's competency in treatment planning. There exists both controllable and uncontrollable variability in our current processes, which would benefit from quality improvement initiatives.

## AUTHOR CONTRIBUTIONS

**Dominic DiCostanzo**: Conceptualization; methodology; software; formal analysis; investigation; data curation; writing—original draft, visualization. **Theodore Allen**: Methodology; formal analysis; writing—review and editing; visualization; supervision. **Ahmet Ayan**: Methodology; data curation; writing—review and editing; supervision. **Allison Quick**: Methodology; writing—review and editing; supervision. **Kevin Evans**: Writing—review and editing; supervision. **Emily Patterson**: Conceptualization; methodology; formal analysis; writing—review and editing; visualization; supervision.

## CONFLICT OF INTEREST STATEMENT

No, there is no duality of interest that I should disclose, having read the above statement.

## ETHICS STATEMENT

This study was approved by our Institutional Review Board.

## Supporting information



Supporting information

## Data Availability

Authors will share data upon request to the corresponding author.
