# Supplementary material for: An assessment of skill erosion on high‐dose rate brachytherapy treatment planning
Source: J Appl Clin Med Phys. 2025 Oct 14;26(10):e70300. doi: 10.1002/acm2.70300 (PMC12521045; doi:10.1002/acm2.70300)
Supplement: Supplementary file 1 — Supporting information [file ACM2-26-e70300-s001.docx]

Supplemental Information

## Conversion of Indices

In support of the analysis, each of the (X, Y, Z) coordinates for both the dwell positions and contour points were converted to XYZ continuous indices and continuous indices of the CT image. The XYZ-indices refer to the conversion of the (X, Y, Z) coordinate to its corresponding index:

$$X=\{X\in\mathbb{R}|0\leq X \leq511\}$$

$$Y=\{Y\mathbb{\in R}|0\leq Y \leq511\}$$

$$Z=\{Z\mathbb{\in R}|0\leq Z \leq511\}$$

$$I=\{I\mathbb{\in R}|0\leq I \leq N\}$$

where $I$ corresponds to the continuous index of the complete CT image and $N$ is the total number of voxels in the image minus 1 due to zero-indexing. Using continuous rather than discrete (integer) indices allows for the accurate representation of spatial points within the image that do not correspond to the center of image voxels.

Conversion of the (X, Y, Z) coordinates to a single-valued continuous index designates each voxel a single number to represent its location in the image. For instance, the first voxel of a 3D volumetric image has an indexed location (0, 0, 0) but a continuous index of 0. For a typical CT scan slice consisting of a 512x512 matrix, there are 262,144 voxels, with the last voxel having a continuous index of 262,143, as the indices are 0-indexed. However, converting physical coordinates and indices requires knowledge of the image's origin, spacing, direction, and rotation. The following formula can be used to convert from physical location to index:

$$I=\frac{\left( p-o \right)}{\left( s\cdot d \right)\cdot r}$$

where $I$ is the index being calculated, *p* are the physical point coordinates in 3D space to convert, *o* are the origin coordinates for the image, *s* is the image spacing, *d* is the image direction, and *r* is the image. For CT scans used in treatment planning, the image rotation is generally unity. This calculation must occur in each dimension (X, Y, Z) for a given 3D point. Each component of the calculation can be read from the DICOM header of a CT slice instance. SimpleITK was used to calculate the indices to confirm the calculations. To calculate the continuous index, rather than the coordinate-based indices, the following equation was used:

$$I=(z_{i} \cdot N_{x} \cdot N_{y})+ x_{i} \cdot N_{x}+y_{i}$$

where $x_{i},y_{i},z_{i}$ are the corresponding XYZ-indices and $N_{x},N_{y},N_{z}$ are the size of the image in each dimension. The code for this conversion was implemented in Python, which performs operations in row-major order. The first term corresponds to the calculation of the number of indices in each slice of the image before the current slice, the second term is the row index of the point to convert ${(x}_{i})$ multiplied by the number of voxels in each row, and the final term is the columnar location of the voxel.

## Polynomial Fits

The fit was performed using the *Polynomial.polyfit* function of Numpy v1.24.1 (<https://numpy.org/>). The tandem points were fit with a quadratic polynomial to support situations where the dwell positions extended into the bend of the tandem. If the tandem was contoured with two points, then the squared term of the quadratic was set to zero to create a linear fit. The ring was fit with a 4^th^-order polynomial. [See Figure S1]


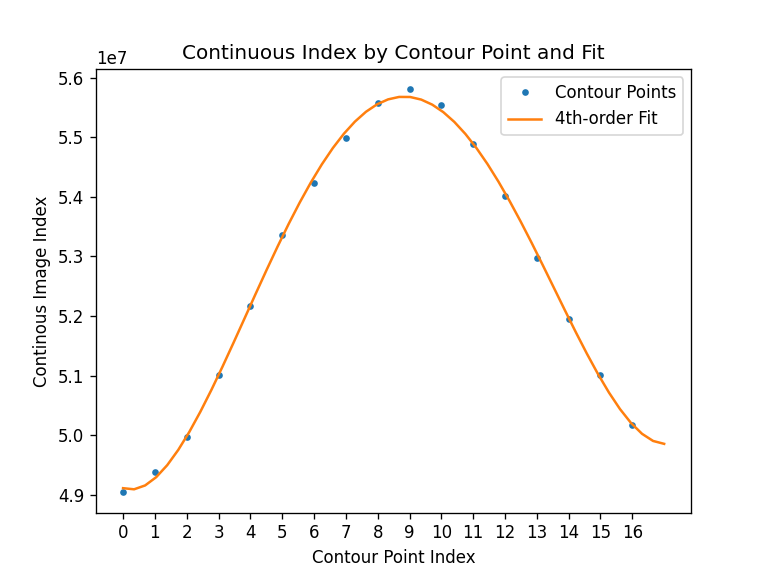


Figure S1: An example of the 4^th^-order fit used to identify the variability of the ring reconstruction. The orange line is the fit as determined by the contour points manually selected [blue dots]. Notice the minor deviations for indices 1, 7, 9, and 10. These differences manifest as a lower coefficient of determination allowing use as a surrogate for the accuracy of the ring contour. A coefficient of determination of 1.0 would indicate perfect agreement of the fit with the contour, this also would indicate that the individual reconstructing the applicator created a smooth applicator consistent with the physical applicator inserted into a patient.
